# Thematic analysis of a United Kingdom-wide survey to explore women’s perceptions and concerns about assisted reproductive technology

**DOI:** 10.1080/14647273.2026.2666204

**Published:** 2026-05-12

**Authors:** Faiza Afzal, Ling Yin Fritz Wong, Mitana Purkayastha, Yan Lu, Philippa Rees, Melissa A. Richard, Carrie L. Williams, Philip J. Lupo, Barbara Luke, Alastair G. Sutcliffe

**Affiliations:** a Population, Policy and Practice Department, University College London Great Ormond Street Institute of Child Health, London, UK; b University College London Medical School, London, UK; c Section of Hematology-Oncology, Department of Pediatrics, Baylor College of Medicine, Houston, Texas, USA; d North East London NHS Foundation Trust, London, UK; e Department of Obstetrics, Gynecology, and Reproductive Biology, College of Human Medicine, Michigan State University, East Lansing, Michigan, USA

**Keywords:** Survey, qualitative study, assisted reproductive technology, fertility treatment, health information, educational outcomes, national database

## Abstract

Women’s perceptions of long-term outcomes of assisted reproductive technology (ART) remain underexplored in the United Kingdom. National database studies investigating these outcomes may provide clearer information. This survey investigates women’s perceptions of: long-term ART outcomes, information provision on these outcomes, and such national database studies. Over an 8-month period, women who had undergone ART, were considering ART, or had conceived naturally completed an anonymous, cross-sectional survey distributed via social media. Descriptive and inductive, semantic thematic analyses were performed. Of 562 respondents, most were aged 25–40 (72.4%) and underwent private ART (37.9%). Most reported no concerns about maternal health (51.8%), child health (66.9%) and child education (82.5%). Reported concerns focused on maternal reproductive, cancer and endocrine outcomes, and child reproductive, neurodevelopmental, developmental and learning outcomes. Information on long-term outcomes was frequently not provided by fertility clinics (up to 91.9%). While up to 68.3% considered national database studies useful for investigating offspring outcomes, some raised data confidentiality concerns. Dissemination via healthcare professionals sensitively pre-treatment was preferred. Overall, most women reported no concerns about and received no information on long-term ART outcomes in maternal health, child health and child education. Improving clinician-led information provision may address knowledge gaps and support research dissemination.

## Introduction

Over the last 40 years, the use of assisted reproductive technology (ART), defined as ‘all treatments or procedures that include the *in vitro* handling of both human oocytes and sperm, or embryos, for the purpose of establishing a pregnancy’ (Zegers-Hochschild et al., 2009), has steadily increased. ART procedures include *in vitro* fertilisation (IVF) and intra-cytoplasmic sperm injection (ICSI), but do not include intrauterine insemination (IUI). Approximately 12 million children were reported to have been born after ART worldwide (eClinicalMedicine, 2023), including over 300,000 ART-conceived children in the United Kingdom (UK), with numbers increasing year-on-year (Human Fertilisation & Embryology Authority, 2025). This is partially due to the prevailing social trend amongst couples towards delaying their first planned pregnancy, but could also reflect the impact of lifestyle factors and underlying chronic health conditions (Glazer et al., 2017). However, data on long-term outcomes of ART remain scarce (Pinborg et al., 2023) and are summarised below.

### Maternal health, child health and child educational outcomes following ART

Regarding maternal health, a meta-analysis has shown that ART pregnancies carry a higher risk of *short-term* obstetric complications, including pregnancy-induced hypertension, gestational diabetes and placental abruption, compared to natural conception (Qin et al., 2016). For *long-term* outcomes, however, the risk of overall breast and endometrial cancers is not increased in ART patients compared to the general population, while the higher risk of ovarian cancer found in ART patients is limited to women with endometriosis and/or low parity (Williams et al., 2018). Literature reporting the metabolic, endocrine and inflammatory sequelae of IVF hormonal therapies remains lacking compared to natural conception (Coussa et al., 2020).

Regarding child health, the *short-term* health implications following ART are also well-recognised: ART-conceived children have higher rates of hospital admissions (Sutcliffe et al., 2023), prematurity (Wisborg et al., 2010) and congenital malformation (Qin et al., 2016), and lower birth weight than naturally-conceived peers. However, the potential *long-term* health outcomes are yet to be described definitively. For reproductive outcomes, some data suggest reduced sperm counts in ICSI-conceived male offspring, while no adverse effects were observed in female offspring (Berntsen et al., 2019). However, this potential sex-specific risk is difficult for individuals contemplating ART to take into consideration, at least in the UK, where the sex of the embryo is typically undisclosed during fertility treatment (Fox, 2009). Data reporting neurodevelopmental outcomes were similarly conflicted: associations with ART were often subgroup-dependent or disappeared after adjusting for multiple pregnancies (Berntsen et al., 2019). Furthermore, it is unclear whether these potential adverse outcomes result from ART itself or from the underlying infertility (‘chicken-or-egg dilemma’) (Berntsen et al., 2019; Graham et al., 2022). Understandably, such inconclusive evidence can be difficult for prospective ART patients to interpret, adding further ambiguity and concern to already complex fertility decisions that balance personal hopes with fears for the child’s long-term health.

Regarding child education, four systematic reviews (Bay et al., 2013; Hart & Norman, 2013; Middelburg et al., 2008; Rumbold et al., 2017) found no significant differences in cognitive or neuro-developmental outcomes between ART-conceived and naturally-conceived children. However, the impact of these findings was reduced both by methodological limitations of the underlying studies and by conflicting findings among high-quality studies comparing children conceived with ICSI and IVF (Rumbold et al., 2017).

Considering the above long-term outcomes, it is important to investigate women’s perceptions and potential concerns about ART. In our team’s 2006 survey, only a small proportion of families with ART-conceived children expressed concerns about their child’s future fertility (8%) and general health (3.6%), with even fewer concerned about their child’s educational potential (1%) (Fisher-Jeffes et al., 2006). These align with previous findings showing overall low levels of maternal concern about a child’s vulnerability to illness, although IVF mothers reported significantly higher concern levels than naturally conceiving mothers (Gibson et al., 2000). To our knowledge, no other recent studies have specifically examined women’s concerns about the long-term health and education of ART-conceived children.

### Information provision on ART-related maternal and child outcomes

It is of interest to explore how information on the above long-term outcomes is presented to women at fertility clinics. The UK’s fertility treatment regulator, the Human Fertilisation and Embryology Authority (HFEA), has specified in their Code of Practice that fertility clinics should inform women seeking treatment of ‘the potential immediate and *longer-term* risks of the treatment and any treatment add-ons used, including the risks to the *patient* and the possibility of any *children* conceived having developmental and birth defects’ (Human Fertilisation & Embryology Authority, 2023a). However, little data exist on how these long-term maternal and offspring outcomes are presented in the UK. Current regulatory guidance on information provision at clinics also appears more focused on success rates and short-term outcomes than long-term health or educational outcomes. Insufficient information provision has been shown to undermine patient trust and satisfaction with their ART clinic (Barrière et al., 2019).

### Usefulness and dissemination of national database studies on ART-related child health and educational outcomes

The HFEA has maintained a register of all fertility treatments and outcomes in the UK since 1991. This national database enables data linkage with other datasets, such as Hospital Episode Statistics (Purkayastha et al., 2021) and National Pupil Database, to identify potential long-term ART outcomes on child health and education. Examples include our team’s database studies to investigate the general health outcomes of women undergoing fertility treatment and their ART-conceived children (LIFT Research Group, 2022), as well as the educational outcomes of ART-conceived children (ENCHANT Research Group, 2022). Published findings from these studies can support evidence-based information for ART patients and inform national policy. However, little is known about women’s perceptions of these studies. While this survey forms part of our team’s wider patient and public involvement (PPI) strategy to inform the methodology of our LIFT and ENCHANT studies, it also aims to explore patient perceptions of the usefulness and dissemination of national database studies investigating ART outcomes in child health and education.

### Aims and objectives

In this survey, we aim to explore women’s perceptions and concerns about ART through the following objectives:

1. To describe participants’ demographic characteristics and experiences with fertility treatment (Domain 1)
2. To explore participants’ concerns, if any, about the long-term ART outcomes regarding maternal health, child health and child education (Domain 2)
3. To assess participants’ views on the quality of information provision by fertility clinics on long-term ART outcomes regarding maternal health, child health and child education (Domain 3)
4. To understand participants’ perceptions of the usefulness and dissemination of national database studies investigating ART-related child health and educational outcomes (Domain 4)

## Methods

### Participants

Participants were identified by convenience (volunteer) sampling, where participants volunteered to take part in the survey. Women who underwent ART, were considering ART, or conceived naturally were invited to participate.

### Survey design and distribution

The cross-sectional, English, online survey was designed and hosted on Qualtrics. It was distributed via Fertility Network UK’s social media channels (Instagram, Facebook and Twitter/X), which collectively reach over two million users annually. The charity provides support for people with fertility issues across the UK and has over 57,000 members in its support groups (Fertility Network UK, c.2025). No paid advertisements were used. To extend visibility, the survey link was also shared with our broader research network by being pinned on the websites of our larger studies (ENCHANT Research Group, 2022; LIFT Research Group, 2022).

A background information page was provided before displaying the survey questions. This outlined the survey’s rationale, target audience and examples of our team’s national database studies for context. We broadly referenced the intended methodology of our national database studies, without specifying the study name, to explore perceptions more generally and avoid bias (Perrotta et al., 2025). Participants were not asked about their willingness to participate or to compare database studies with other research designs. The ensuing survey collected data across four domains (Table 1) to address our objectives as described previously in the Introduction. All survey questions were optional. The survey was anonymous and did not capture any personal identifiable data. The survey was active for 8 months from 14 July 2023 to 14 March 2024.

### Data analysis

Qualitative analysis of free-text responses (Q3–14) followed Braun and Clarke’s (2006) thematic analysis using an inductive, semantic and post-positivist approach, whereby themes were derived from the data, reflected the explicit meaning of responses, and triangulated through team discussion and quantitative findings, respectively. Each phase of the analysis was further guided by Nowell et al.’s (2017) approach to establish trustworthiness, as detailed below. LYFW manually coded all responses on NVivo 14 and connected the codes into broader themes and sub-themes. The resulting codebook was independently reviewed by FA and discussed in monthly team meetings involving the core researchers (FA, LYFW, MP and YL) to gather feedback and achieve consensus on the coding. This review process was repeated 5–7 times for each open-ended question to iteratively revise the themes, with reflexive memos and audit trails documented throughout. Revisions primarily aimed to improve clarity and ensure accurate representation of the responses. Code frequencies were counted to derive percentages for comparison across questions, and illustrative quotes were identified to support our findings. In our survey design, some questions were not applicable (N/A) to certain respondents, e.g. questions on treatment year or provider (Q8, 9) did not apply to respondents who were still considering treatment (Q1), questions on information provision regarding the ART-conceived child’s health or educational outcomes (Q4, 6) did not apply to those with unsuccessful childbirth (Q2). Therefore, some participants responded with ‘N/A’ and these responses were excluded from the analysis using pairwise deletion as they did not provide meaningful qualitative data under our semantic approach (Byrne, 2022).

Quantitative analysis of categorical responses (Q1, 2 and 15–17) was performed to produce descriptive statistics. Due to cells with zero values and uneven distributions in respondent characteristics, Fisher’s exact test was used to compare group differences in these characteristics, with statistical significance set at two-tailed p < 0.05. Sensitivity analyses excluding ‘Did not answer’ (i.e. missing responses) or ‘N/A’ responses were performed to assess the impact of survey completion on the findings. All analyses were conducted on StataNow/MP 18.5.

### Ethical approval and consent

Ethical approval was obtained from University College London’s Research Ethics Committee (28371/001). A disclaimer was added to the participant information sheet with contact details of Fertility Network UK. This was to ensure that participants were provided adequate support if they were concerned or affected by any survey questions. All participants provided informed consent to participate in the research.

## Results

Of the 1273 times the survey was accessed, 575 responses were submitted. Thirteen submissions provided empty answers to all questions and were excluded; therefore, 562 participants were included for analysis (completion rate 44.1%). As participation in each question was optional, the total number of responses varied across questions (Table 1; mean ± SD 454 ± 91 responses) and only Q1 achieved complete response. Item response rates were highest in Domain 1 (mean 95.7%) and lowest in Domain 4 (mean 59.4%). Themes are presented in the following headings, and sub-themes are presented in Tables 3–5.

### Domain 1: Demographics and experience with fertility treatment

Most respondents were 25–40 years old (72.4%), White (90.2%) and held a university degree or higher qualification (84.9%) (Table 2). A significant difference was found between age group and fertility treatment stage (p = 0.012), with the highest ART engagement among women at the typical reproductive age of 25–40. Ethnicity and education did not differ significantly across treatment stages.

Most fertility treatments were undertaken in the 2020s (62.8%), and over half resulted in successful childbirth (52.3%). Around one-third of respondents underwent fertility treatment in the private sector (37.9%) and a similar proportion through the public National Health Service (NHS) (31.9%). Of the 19.9% who attended both, some switched from NHS to private treatment (n = 34), mentioning push factors such as lack of funding (*‘One cycle NHS, then funding was revoked in the area and went privately’*, P98) and failed rounds (*‘Initially NHS clinic unsuccessfully and then private clinic successfully’*, P140). Few switched from private to NHS (n = 6): *‘First private for IUI then NHS for IVF’* (P106). As expected, women who had undergone treatment had different ART-specific experiences, including childbirth following ART, treatment year and treatment provider (all p < 0.001), compared to those only considering treatment or who had conceived naturally.

Sensitivity analyses excluding ‘Did not answer’ or ‘N/A’ responses showed no change in significance across all questions in Table 2. Questions on fertility treatment (missingness: 11.0% in Q8 and 8.9% in Q9) were generally skipped more frequently than demographic questions (2.8–3.4%) (Table 2). As expected, over half of the women who were considering fertility treatment skipped questions related to receiving fertility treatment (59.7% in Q8 and 53.2% in Q9).

### Domain 2: Uncommon but diverse concerns about ART outcomes in maternal health, child health and child education

**Figure 1.**
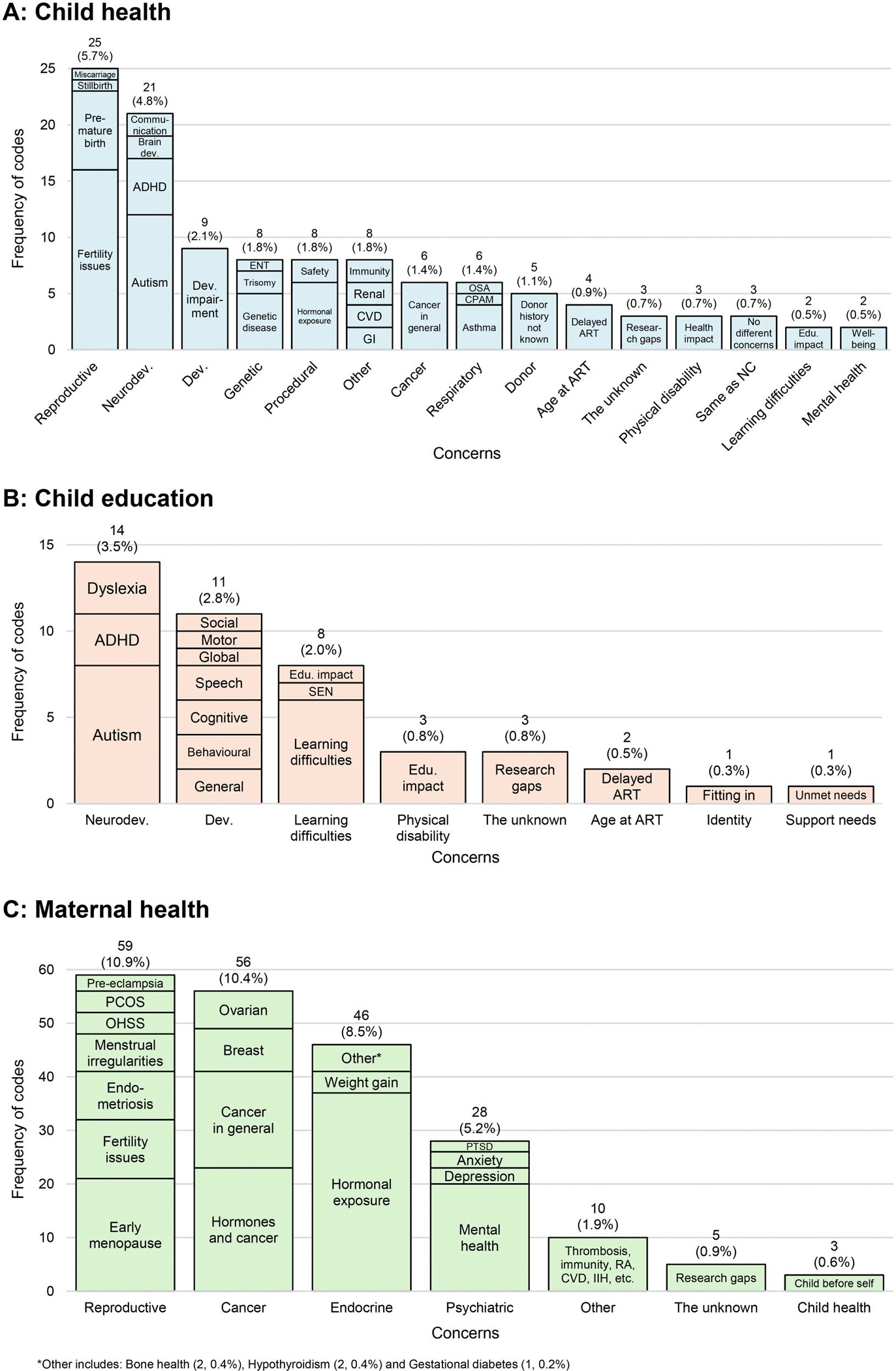
Respondents’ concerns about long-term ART outcomes in **(A)** child health, **(B)** child education and **(C)** maternal health. Labels within bars represent codes produced from thematic analysis, and the labels above bars represent the total frequency and percentage of codes. Percentage values do not total 100% because ‘N/A’ and single-word responses without elaboration (e.g. ‘Yes’, ‘Some’ and ‘Possibly’) did not provide meaningful data and thus were excluded. **Abbreviations:** ADHD, attention deficit hyperactivity disorder; CPAM, congenital pulmonary airway malformation; CVD, cardiovascular diseases; Dev., development; Edu., education; ENT, ear, nose and throat; GI, gastrointestinal; IIH, idiopathic intracranial hypertension; NC, natural conception; Neurodev., neurodevelopment; OHSS, ovarian hyperstimulation syndrome; OSA, obstructive sleep apnoea; PCOS, polycystic ovarian syndrome; PTSD, post-traumatic stress disorder; RA, rheumatoid arthritis.

Overall, almost half of respondents (48.6% in Q10) had not considered potential short- and long-term health outcomes of ART. In particular, over half (51.8% in Q11, 66.9% in Q3 and 82.5% in Q5) reported no concerns about its long-term outcomes with regard to: respondent’s own health (*‘No, perhaps I’m blissfully unaware’*, P16, age 25–40, unsuccessful fertility treatment); child’s health (*‘No, at the moment I just want a child’*, P237, age 25–40, considering fertility treatment); and child’s education (*‘Not at all, she is doing very well at school and is in year 7*′, P176, age 40–60, successful fertility treatment), listed from most to least concerned. The breadth of concerns expressed by the remaining respondents is shown in Figure 1, with the top three concerns summarised in Table 3.

Regarding maternal health, the most common concerns were related to women’s reproductive outcomes, cancer risks and endocrine effects. The impact of hormonal treatment emerged as a recurring theme underpinning the above concerns (Table 3).

Regarding child’s health, participants most frequently raised concerns about child reproductive, neurodevelopmental and developmental outcomes. A sense of guilt, worry and uncertainty, particularly around the perceived generational implications of passing on fertility issues, was reflected in participant quotes (Table 3): *‘They could possibly have the same fertility issues as us, their parents’* (P446, age 25–40, considering fertility treatment).

Regarding child’s education, concerns were mainly about neurodevelopmental outcomes, child development and learning difficulties. Autism and attention deficit hyperactivity disorder were reported among the more common concerns in child health and education (Figures 1A–1B).

Of the participants who did not consider potential short- and long-term ART outcomes (48.6%), 14 explained that they believed the benefits of ART outweighed the risks. Other respondents had considered short-term (7.2%) more than long-term (4.4%) health outcomes. Short-term outcomes considered included hormonal exposure, ovarian hyperstimulation syndrome and weight changes; long-term outcomes considered included mental health, reproductive health and cancer. Sixteen participants noted concerns arose after treatment, eight during treatment and six before treatment. Participant quotes suggested these concerns may reflect the perceived adequacy of information provision (Table 3).

### Domain 3: Information on long-term ART outcomes was rarely presented by fertility clinics to women

Overall, most respondents reported that fertility clinics did not provide information on health and educational outcomes following ART and that, when provided, healthcare professionals or videos were the most common channels of communication. Most participants said that no information about child health (75.2%) and educational outcomes (91.9%) was presented. As a result, some participants had to conduct their own independent research for self-education (Table 4). Only a small proportion (<10%) reported that such information was provided by healthcare professionals and videos. Furthermore, 56.6% of participants said that no information was provided about their own health outcomes following fertility treatment: *‘I have no idea what I should be worried about, my private fertility clinic never explained any risks or research findings with me’* (P412, age 25–40, unsuccessful fertility treatment).

### Domain 4: Overall positive perceptions of the usefulness and dissemination of national database studies on ART outcomes in child health and education

More than half (54.8%) had positive views about researchers accessing existing large national datasets to retrieve and analyse information about children’s health and educational outcomes: *‘I agree with the methodology. Analysing anonymous data is a good option to ensure privacy of patients’* (P1, age 25–40, successful fertility treatment) (Table 5). Some had negative perceptions (13.2%) or concerns (5.5%). In terms of the use of large patient datasets to retrieve information, a higher proportion (68.3%) had positive perceptions of its usefulness: *‘Studies like these will help those considering fertility treatment in the future’* (P50, age 25–40, successful fertility treatment). Others had negative perceptions (12.5%) or conditional perceptions related to data confidentiality (3.2%). Regarding dissemination of findings, participants expressed that study findings should be disseminated prior to treatment (7.8%), by healthcare professionals (6.5%) and sensitively (4.5%), e.g. *‘People should be informed of potential risks when they have their first appointment with IVF clinic and further discussion should be offered’* (P86, age 25–40, successful fertility treatment).

## Discussion

### Main findings

To our knowledge, this is the first large-scale qualitative survey exploring the perceptions and concerns about ART among women. A key finding of this study is that most participants reported no concerns about the long-term outcomes of ART with regard to their own health or their child’s health and education, and interestingly, these women reported that they had received no information on these issues from clinics. Receiving limited information from clinics could prevent women from recognising potential ART outcomes that may warrant concern. The high proportion of participants reporting no concerns in this study is consistent with the results of our previous survey (Fisher-Jeffes et al., 2006). Other studies have found that most fertility treatment patients hold positive attitudes about ART (Schmidt et al., 2003; Szalma & Bitó, 2021). Meanwhile, considering it has been reported that information provision is identified by patients as their top priority in patient-centred infertility care (Dancet et al., 2011), this identifies an unmet information need in both our sample and the wider literature. Wilkinson et al. (2020) have stressed that ‘the scientific community has warned of the need to inform patients about the association between ART and offspring health for more than a decade’, but Assaysh-Öberg et al. (2023) more recent meta-ethnographic analysis of 19 qualitative studies shows that women ‘felt uninformed about the long-term effects of treatment, on themselves and on their fetus and possible child’. Therefore, ART clinics have a clear responsibility to inform patients of the offspring and maternal outcomes; it should not be left to patients to conduct their own research (Barnhart, 2013), as seen in participant quotes.

Among those who expressed concerns, those regarding child health focused on reproductive, neurodevelopmental and developmental outcomes, while maternal concerns related to reproductive conditions, cancer risk and hormonal exposure. These findings highlight that, beyond achieving pregnancy, a small number of women undergoing or considering ART are attentive to the long-term implications of the treatment. This is often interwoven with the hope, desperation and unease they experienced during fertility treatment, as reflected in participants’ quotes. Quotes also demonstrated participants’ familiarity with medical terminology, aligning with their high educational background. Concerns about hormonal treatment side-effects (Barrière et al., 2019) and reproductive conditions (Leyser-Whalen et al., 2022) in women are well-documented. Conversely, although there is ample literature on potential ART-related child health outcomes (Berntsen et al., 2019), studies exploring women’s *perceptions* of these child health outcomes are limited, as are those exploring women’s perceptions about child educational outcomes. This may suggest such concerns are generally uncommon and thus may be understudied or underreported in literature. Our findings may provide a novel contribution by describing the perceptions of the small proportion of women who expressed concerns in regard to child health and educational outcomes.

Overall, respondents supported the use of national databases to investigate child outcomes and showed a preference for study findings to be communicated by healthcare professionals before treatment, while a few participants raised concerns about data confidentiality. The literature has consistently emphasised the importance of the provision of high-quality information (Dancet et al., 2010) with transparency (Perrotta et al., 2025), sensitivity (Holter et al., 2021) and empathy by doctors (Malin et al., 2001), aligning with respondents’ preferences in our study.

### Strengths and limitations

This study’s strengths include its large UK-wide sample and comprehensive scope across short- and long-term health and educational outcomes. This is in comparison to other qualitative surveys conducted on a similar population and setting (Boivin et al., 2020; Harrison et al., 2021; Sousa-Leite et al., 2023). The use of predominantly free-text questions yielded richer and more diverse qualitative insights than structured questions allow (O’Cathain & Thomas, 2004), helping to address a gap in ART research that often prioritises clinical success over patient experience (Pennings & Ombelet, 2007).

Nonetheless, limitations should be considered when interpreting the results. Voluntary participation may have introduced self-selection bias (Bethlehem, 2010), with individuals who had more adverse experiences being more likely to respond to the optional open-ended questions and thus being potentially overrepresented (Redshaw et al., 2007). Recruitment was primarily conducted through a single national UK charity, which may limit representativeness despite its wide social media reach and high engagement with the UK fertility patient community. Our predominantly White, highly educated sample may further limit generalisability, though this reflects patterns reported in other qualitative ART studies (Li et al., 2025; Mayette et al., 2024; Redshaw et al., 2007; Rothwell et al., 2020). Similarly, the age distribution of our respondents and the proportion who had undergone NHS-funded fertility treatment are comparable to those of the UK fertility patient population; however, the proportion of White respondents and the rate of successful fertility treatment in our sample are moderately higher than the national population (Human Fertilisation & Embryology Authority, 2023b). Our UK-based findings may not be generalisable across other countries due to social and financial differences surrounding ART (Dancet et al., 2010). The cross-sectional design captures perceptions at a single time point and cannot determine how views may change throughout fertility treatment and thereafter. Thematic analysis involves subjective interpretation, although observer bias was minimised through researcher triangulation and peer debriefing (Nowell et al., 2017). All survey questions were optional, and missing responses may introduce non-response bias. Although over half of those who responded viewed national database studies as useful, item response rates for questions referring to this issue were relatively low, so the extent of positive perceptions should be interpreted with caution. The use of skip logic in survey design could have directed respondents to the relevant questions, potentially reducing the number of missing or ‘N/A’ responses and improving the survey completion rate. Finally, including men’s or couple’s perspectives may offer useful comparisons with women’s views, but this was beyond the scope of this study and has been addressed in our separate survey exploring men’s perceptions (Afzal et al., 2026).

### Implications for clinical practice

Women’s concerns about long-term outcomes of ART, albeit expressed by a small proportion, and their perceived lack of information provision highlight the need for patient education and patient-centred communication. Alongside the top concerns identified in this survey, validated ART questionnaires such as the Concerns During Assisted Reproductive Technologies (CART) scale (Klonoff-Cohen & Natarajan, 2004) can inform clinicians about individual concerns and help tailor the content of information delivered. Information provision has been shown to reduce treatment-related concerns (Gameiro et al., 2013), and formalising this through online education programmes for ART users, such as clinic-led video-based modules with comprehension assessments on common ART procedures and medications (Bernard et al., 2022; Jones et al., 2020), can improve psychological outcomes while offering a cost-effective solution for clinics (Cousineau et al., 2008). Overall, the literature supports both the feasibility and effectiveness of patient education in addressing women’s concerns.

It is acknowledged that from providers’ perspectives, addressing women’s concerns amid inconclusive evidence can be challenging. Detailed explanations for communication challenges have been explored elsewhere (Klitzman, 2018). In this study, healthcare professionals were the primary source of information, when it was presented, and it is important that their communication with patients must balance transparency and sensitivity. National Institute for Health and Care Excellence (2013) clinical guidelines offer clear recommendations on discussing long-term ART safety, including areas where evidence is still awaited. Although fear and anxiety were expressed by only a small proportion of respondents, provision of emotional support by fertility counsellors, clinicians and psychologists (Dancet et al., 2011), targeted for those patients, may help to alleviate uncertainty through patient-centred communication.

### Implications for future research

Women’s concerns about long-term outcomes, or the widespread lack of concern which may reflect limited information, emphasises the need for further national database studies, an approach broadly supported by respondents. These qualitative insights will be used to inform our national data linkage studies and guide how we disseminate their findings to key stakeholders. In line with respondents’ preferences, our results would be shared sensitively with patients, via healthcare professionals where appropriate, and ideally prior to treatment. As demonstrated in this survey and reported in the wider literature, incorporating PPI into ART studies can guide research priorities and build public trust. Trust is crucially important in fostering patient engagement with data use (Carson et al., 2019), and PPI has been shown to meaningfully impact the direction of laboratory (Fleming et al., 2021) and clinical research (Healey et al., 2024). Additionally, qualitative longitudinal studies with focus groups or interviews could provide deeper insights into how women’s views may evolve at different treatment stages. Considering the known racial disparities in fertility research shaped by cultural and systemic factors (Ekechi, 2021), future studies should consider broadening representation of Black, Asian and Minority Ethnic (BAME) populations by using multiple recruitment sources, translated survey materials and partnership with community liaison workers (Farooqi et al., 2022). These strategies would facilitate culturally competent and inclusive co-production with ethnic minority communities, enabling meaningful input into future study methodology and dissemination.

## Conclusion

Most women who responded reported no concerns about, or that they had received no information on long-term ART outcomes in maternal health, child health and child education, while some raised important concerns about maternal and offspring outcomes. Overall, participants supported the use of national databases to investigate these outcomes, highlighting the need for appropriate dissemination, patient-centred communication and PPI-informed research that reflects their requirements and concerns.

## Figures and Tables

**Table 1.** Domains and item response rates of survey questions.

Domain	Survey questions	Responses (item response rate)

1. Demographics and experience with fertility treatment	Q1: Are you considering fertility treatment, have undergone fertility treatment or have conceived naturally?	562 (100.0%)
	Q2: Have you had children following assisted reproductive technology (ART)?	561 (99.8%)
	Q8: If you have undergone fertility treatment, how long ago was it?	500 (89.0%)
	Q9: Was it at a Private or NHS clinic?	512 (91.1%)
	Q15: What age group category applies to you?	546 (97.2%)
	Q16: What is your ethnic group?	544 (96.8%)
	Q17: What is your educational background?	543 (96.6%)
2. Concerns about health and educational outcomes	Q3: Do you have any particular concerns about the long-term health outcomes of your child/children? If yes, what are your concerns?	416 (74.0%)
	Q5: Do you have any particular concerns about the long-term educational outcomes of your child/children? If yes, what are your concerns?	398 (70.8%)
	Q10: Were potential short- and long-term health outcomes something you considered before/during/after fertility treatment?	512 (91.1%)
	Q11: Do you have any particular concerns regarding long-term health outcomes after fertility treatment?	495 (88.1%)
3. Information provision at fertility clinics	Q4: How was the information on your ART child's health outcomes presented to you (if at all)?	351 (62.5%)
	Q6: How was the information on your child's educational outcomes presented to you (if at all)?	334 (59.4%)
	Q12: How was information on your long-term health outcomes (following ART) presented to you (if at all)?	445 (79.2%)
4. Perceptions of the usefulness and dissemination of national database studies	Q7: What are your thoughts about analysing large anonymous data sets about your child's health and educational outcomes?	306 (54.4%)
	Q13: What are your thoughts about using large administrative patient data sets to retrieve information?	349 (62.1%)
	Q14: What are your views on how the findings of these studies could/ should be presented to people undergoing fertility treatment?	347 (61.7%)

Abbreviations: ART, assisted reproductive technology; NHS, National Health Service; Q, question number.

**Table 2.** Demographic and fertility treatment characteristics of respondents (Domain 1).

Respondent characteristics Demographics		Stage of fertility treatment (Q1, 562 responses)		Total (n = 562) *n* (%)	Group differences *p*-value

	Undergone fertility treatment (n = 496) *n* (%)	Considering fertility treatment (n = 62) *n* (%)	Conceived naturally (n = 4) *n* (%)		

**Age group (Q15, 546 responses)**					0.012*
Under 25	1 (0.2)	0	0	1 (0.2)	
25-40	352 (71.0)	52 (83.9)	3 (75.0)	407 (72.4)	
40-60	131 (26.4)	7 (11.3)	0	138 (24.6)	
Did not answer	12 (2.4)	3 (4.8)	1 (25.0)	16 (2.8)	
**Ethnic group (Q16, 544 responses)**					0.051
White	451 (90.9)	54 (87.1)	2 (50.0)	507 (90.2)	
Asian/Asian British	14 (2.8)	3 (4.8)	0	17 (3.0)	
Black/Black British/Caribbean/African	8 (1.6)	1 (1.6)	0	9 (1.6)	
Mixed or multiple ethnic groups	5 (1.0)	0	0	5 (0.9)	
Other ethnic groups	4 (0.8)	1 (1.6)	1 (25.0)	6 (1.1)	
Did not answer	14 (2.8)	3 (4.8)	1 (25.0)	18 (3.2)	
**Highest educational qualification (Q17, 543 responses)**					0.145
No formal qualifications	3 (0.6)	0	0	3 (0.5)	
GCSE's or equivalent	16 (3.2)	5 (8.1)	0	21 (3.7)	
A Levels or equivalent	40 (8.1)	2 (3.2)	0	42 (7.5)	
Degree or equivalent	244 (49.2)	29 (46.8)	1 (25.0)	274 (48.8)	
Higher degree or equivalent	179 (36.1)	22 (35.5)	2 (50.0)	203 (36.1)	
Did not answer	14 (2.8)	4 (6.5)	1 (25.0)	19 (3.4)	
**Experience with fertility treatment**	*n* (%)	*n* (%)	*n* (%)	*n* (%)	*p*-value
**Childbirth following ART (Q2, 561 responses)**					<0.001*
Yes	293 (59.1)	1 (1.6)	0	294 (52.3)	
No	203 (40.9)	60 (96.8)	4 (100.0)	267 (47.5)	
Did not answer	0	1 (1.6)	0	1 (0.2)	
**Earliest year receiving fertility treatment (Q8, 500 responses)** ^†^					<0.001*
1990-1999	3 (0.6)	0	0	3 (0.5)	
2000-2009	13 (2.6)	0	0	13 (2.3)	
2010-2019	138 (27.8)	0	0	138 (24.6)	
2020-2024	318 (64.1)	7 (11.3)	2 (50.0)	327 (58.2)	
Not yet	0	7 (11.3)	1 (25.0)	8 (1.4)	
N/A^‡^	0	11 (17.7)	0	11 (2.0)	
Did not answer	24 (4.8)	37 (59.7)	1 (25.0)	62 (11.0)	
**Most recent year receiving fertility treatment (Q8, 500 responses)** ^†^					<0.001*
1990-1999	3 (0.6)	0	0	3 (0.5)	
2000-2009	11 (2.2)	0	0	11 (2.0)	
2010-2019	114 (23.0)	0	0	114 (20.3)	
2020-2024	344 (69.4)	7 (11.3)	2 (50.0)	353 (62.8)	
Not yet	0	7 (11.3)	1 (25.0)	8 (1.4)	
N/A^‡^	0	11 (17.7)	0	11 (2.0)	
Did not answer	24 (4.8)	37 (59.7)	1 (25.0)	62 (11.0)	
**Healthcare sector providing fertility treatment (Q9, 512 responses)**					<0.001*
Private	207 (41.7)	6 (9.7)	0	213 (37.9)	
NHS	164 (33.1)	13 (21.0)	2 (50.0)	179 (31.9)	
Both private and NHS	109 (22.0)	3 (4.8)	0	112 (19.9)	
• NHS then private (n = 34)					
• Private then NHS (n = 6)					
• Order unspecified (n = 72)					
N/A^‡^	0	7 (11.3)	1 (25.0)	8 (1.4)	
Did not answer	16 (3.2)	33 (53.2)	1 (25.0)	50 (8.9)	

* Significant at p < 0.05.

† Some participants reported undergoing more than one round of fertility treatment.

‡ Some participants put 'N/A' as their response.

**Abbreviations:** ART, assisted reproductive technology; GCSE, General Certificate of Secondary Education; N/A, not applicable; NHS, National Health Service; Q, question number.

**Table 3.** Concerns about child health, child educational and maternal health outcomes following ART (Domain 2). Top three concerns (sub-themes) by code frequency are presented.

Question (total code frequency)	Top three concerns (*n*, % of codes)	Illustrative quotes (participant number)

Long-term health outcomes of child (Q3, *N* = 435)	Reproductive concerns (25, 5.7%)	• 'Will she struggle to conceive also?' (P437) • 'Passing on fertility issues to my children.' (P120) • 'Risk of our son being infertile as he was conceived via IVF with ICSI.' (P182)
	Neurodevelopmental concerns (21, 4.8%)	• 'Initially concerns around risk of autism, as I was told taking progesterone can increase the risk. I was then told it doesn’t, so conflicting information.' (P32) • 'One has microdeletion and the other was TFMR due to ventriculomegaly.' (P114)
	Developmental concerns (9, 2.1%)	• 'One of my twins was born with hemifacial microsomia, currently only 7 months but slightly behind in development currently.' (P198) • 'If they will hit milestones on time or if IVF will cause problems later on in terms of her health.' (P344)
Long-term educational outcomes of child (Q5, *N* = 399)	Neurodevelopmental concerns (14, 3.5%)	• 'As an older mum if I could use my own eggs maybe my child might have autism.' (P249) • 'Perhaps a higher risk of autism (and I could be wrong) however this is not a huge concern.' (P295)
	Developmental concerns (11, 2.8%)	• 'My son is non verbal so obviously I am concerned about how he can access education.' (P388) • 'Due to her chromosome disorder, she will likely have global development delay.' (P203)
	Learning difficulties (8, 2.0%)	• 'Are there implications on learning outcomes for children conceived via IVF and more specifically, using ICSI.' (P261) • 'Yes - worried about child having SEN.' (P271)
Long-term health outcomes of respondent (Q11, *N* = 539)	Reproductive concerns (59, 10.9%)	• 'Yes. I now have endometrial hyperplasia. Potentially endometriosis too. Would take all that in a heartbeat for our girl, but it’s tough.' (P13) • 'Effect of blasting my system with hormones. Worried that it reduced my already limited eggs to nothing with all the stimulation.' (P191)
	Cancer concerns (56, 10.4%)	• 'Yes I worry about my increase particularly female cancers due to all the hormones and drugs.' (P80) • 'Yes I had been hearing and reading about multiple cycles/exposure to hormonal drugs increasing your chances of hormonal cancer.' (P90) • 'I suppose general concerns about cancer but a lot of what is out there about it seems to be fear mongering.' (P138)
	Endocrine concerns (46, 8.5%)	• 'I'm concerned about the amount of hormones I've had. I've had 9 rounds of IVF, and 2 rounds of IUI.' (P252) • 'It is a bit worrying pumping body full of hormones. Who knows what long term outcomes will be.' (P474)
Consideration of short and long-term health outcomes before/during/after fertility treatment (Q10, *N* = 541)	Not considered (263, 48.6%)	• 'Not really. Apart from bloating and being uncomfortable. The level of information provided by the clinic is still astounding. I.e. pretty much nothing.' (P522) • 'Not before, I didn’t realise how much the hormones would affect my health after a failed cycle for as long as they did.' (P504) • 'No, it was the only option to hopefully become parents so we didn’t really think about potential health outcomes, we just looked at hopefully becoming a family.' (P484)
	Considered short-term health outcomes (39, 7.2%)	• 'It was explained at the start of the IVF treatment.' (P532) • 'Yes but very briefly and almost matter of fact.' (P496) • 'Yes, we read up and researched lots of information regarding our treatment.' (P398)
	Considered long-term health outcomes (24, 4.4%)	• 'Yes but there doesn’t seem to be much information on the long term outcomes.' (P501) • 'Just typical maternal health issues around being pregnant in 40s.' (P449)

**Abbreviations:** ICSI, intra-cytoplasmic sperm injection; IUI, intrauterine insemination; IVF, in vitro fertilisation; P, participant number; Q, question number; SEN, special educational needs; TFMR, termination for medical reasons.

**Table 4.** Information provision at fertility clinics on ART-related maternal and child outcomes (Domain 3). Top three information sources (sub-themes) by code frequency are presented.

Question (total code frequency)	Top three information sources (n, % of codes)	Illustrative quotes (participant number)

How information on health outcomes of the ART-conceived child was presented (Q4, *N* = 242)	Not presented (182, 75.2%)	• 'Not presented. I did my own research looking at peer reviewed evidence sources.' (P4) • 'Not mentioned, as far as I'm aware there are no long term implications for our daughter.' (P339) • 'It hasn’t been. Should there be something I know about and don’t?' (P361) • 'It wasn’t - assumed same as natural conception.' (P462)
	Presented via healthcare professionals (23, 9.5%)	• 'Neonatal doctor visited after birth and did a check and GP at 8 weeks. Everyone seems to think if you're a first time mum you're either clueless or overreacting.' (P312) • 'Consultant informed us of possible health risks before we had treatment.' (P333) • 'No info presented until I got pregnant and they scared us with facts about the risks of having an IVF baby. But it has only been about them as a baby, no future concerns about them growing up.' (P20)
	Presented via videos (8, 3.3%)	• 'Information on risks to child in videos as part of consent process.' (P365)
How information on long-term educational outcomes of the ART-conceived child was presented (Q6, *N* = 246)	Not presented (226, 91.9%)	• 'Not at all, I researched about neurodivergence myself.' (P150) • 'There was no specific info related to IVF children and education at all.' (P202) • 'Can’t recall them mentioning this.' (P333)
	Presented via healthcare professionals (2, 0.8%)	• 'At work. I work in the NHS.' (P249)
	Presented via videos (2, 0.8%)	• 'In a video presentation.' (P199)
How information on long-term health outcomes of the respondent was presented (Q12, *N* = 491)	Not presented (278, 56.6%)	• 'Not presented but wouldn’t have made any difference about my decision to have treatment.' (P191) • 'All research was done online - no doctors advised me on this.' (P240) • 'Not really given information about long term effects - I googled a lot myself.' (P357) • 'It wasn’t, there was no real support to handle the disappointment and feelings of failure.' (P445)
	Presented via healthcare professionals (48, 9.8%)	• 'Private - verbally immediately after the diagnostics and then in a follow up diagnostics with a consultant.' (P249) • 'We had an implications appointment before treatment where this was discussed.' (P15) • 'Midwives were great help in having GD and supporting it.' (P408)
	Presented via videos (20, 4.1%)	• 'Videos on side effects as part of consent process - less focus on the long term.' (P365) • 'As part of consent video, we were told of risks of IVF (e.g. ovarian hyperstimulation). We were reassured that cancer risk was no higher for women undergoing IVF but NICE guidelines, and some studies, imply otherwise.' (P549)

**Abbreviations:** ART, assisted reproductive technology; GD, gestational diabetes; IVF, in vitro fertilisation; NICE, National Institute for Health and Care Excellence; P, participant number; Q, question number.

**Table 5.** Perceptions of the usefulness and dissemination of national database studies on ART-related child health and educational outcomes (Domain 4). Top three perceptions (sub-themes) by code frequency are presented.

Question (total code frequency)	Top three perceptions (*n*, % of codes)	Illustrative quotes (participant number)

Perceptions of study usefulness: analysing large anonymous data sets about your child's health and educational outcomes (Q7, *N* = 272)	Positive perceptions of study usefulness (149, 54.8%)	• 'Analysing anonymous data is a good option to ensure privacy of patients.' (P1)
	Negative perceptions of study usefulness (36, 13.2%)	• 'It would be interesting to know what controls are being used. How will you adjust for external factors such as parents genetics, social background of parents etc. i.e. all other things that could have an impact on health and educational outcomes regardless of if a baby was IVF conceived.' (P117)
	Concerns about implications of findings (15, 5.5%)	• 'It’s also scaremongering for people who have had a child through fertility treatment or hope to, that their child may have educational needs that they may not have if conceived naturally.' (P151) • 'I have concerns though regarding the implications of the findings in relation to how they will be published and how it may inform policy. Additionally if concerns are raised is their a duty of care to parents for ongoing support in light of this and has it been considered who will provide this.' (P370)
		• 'Infertility is a huge, emotional issue and to insinuate there may be a negative difference in health and educational outcomes of a child born from fertility treatments is pretty insensitive.' (P540)
Perceptions of study usefulness: using large administrative patient data sets to retrieve information (Q13, *N* = 344)	Positive perceptions of study usefulness (235, 68.3%)	• 'I feel given the concerns with declining fertility rates globally that any research that it needs to consider a large patient data set.' (P179) • 'The larger studies often show better results which can be relied upon but across the board there are only small trials.' (P325)
	Negative perceptions of study usefulness (43, 12.5%)	• 'There has to be a study but feel it needs a conversation too. Please include those who aren’t parents too.' (P9) • 'It’s a very quantitative approach and you could miss out on some important qualitative date. Perhaps a qualitative study on a smaller sample could be used as a follow up study to try and support the outcomes of this study.' (P117)
		• 'Useful, but if an effect is shown you'll need more detailed data on parental/donor ages (at time of treatment and time of pregnancy/birth), medications used, numbers of cycles etc. to be really usable.' (P142)
		• 'That seems an invasion of privacy and I don’t consent for my patient records to be used.' (P364)
		• 'Again, there are so many other factors that could affect short and long term health outcomes that just basing the study on fertility treatment is quite frankly, silly.' (P540)
	Conditional perceptions due to data confidentiality (11, 3.2%)	• 'I'm fine with it as long as it remains confidential, is treated with respect and that the researchers continuously hold in their minds we are individuals with families and lives, not just numbers.' (P370)
Perceptions of study dissemination: how study findings could/should be presented to people undergoing fertility treatment (Q14, *N* = 511)	Prior to treatment or in initial consultation (40, 7.8%)	• 'I think that the findings should be presented as part of initial consultation to people seeking fertility treatment. It is important for a potential patient to have all information so that they can make an informed decision on whether or not to proceed with treatment.' (P226) • 'Should be on HFEA website and risks presented prior to treatment (it wouldn’t have changed my decision to pursue IVF but I may have opted for more mild forms to mitigate the risk).' (P450)
	Via healthcare professionals (33, 6.5%)	• 'This information should be made available to all clinics and hospitals so that they can inform patients who are considering fertility treatment about possible outcomes.' (P1) • 'Available in all fertility clinics public and private as well as via GP.' (P290)
	Sensitively (23, 4.5%)	• 'If there are any significant correlations between fertility treatment and a detrimental impact on subsequent children's lives it needs to be delivered sensitively and carefully, avoiding stigma.' (P121)
		• 'I do think that results regarding the health impacts on the mother and the child needs to be presented with caution and sensitivity, as it could add to their anxiety during fertility rounds.' (P252)

**Abbreviations:** GP, general practitioner; HFEA, Human Fertilisation and Embryology Authority; IVF, in vitro fertilisation; P, participant number; Q, question number.

## Data Availability

The data that support the findings of this study are available on request from the corresponding author, FA. The data are not publicly available as they contain information that could compromise the privacy of research participants.
